# Chikungunya Virus and Central Nervous System Infections in Children, India

**DOI:** 10.3201/eid1502.080902

**Published:** 2009-02

**Authors:** Penny Lewthwaite, Ravi Vasanthapuram, Jane C. Osborne, Ashia Begum, Jenna L.M. Plank, M. Veera Shankar, Roger Hewson, Anita Desai, Nick J. Beeching, Ravi Ravikumar, Tom Solomon

**Affiliations:** University of Liverpool, Liverpool, UK (P. Lewthwaite, T. Solomon); National Institute of Mental Health and Neurological Sciences, Bangalore, India (R. Vasanthapuram, A. Desai); Health Protection Agency, Salisbury, UK (J.C. Osborne, J.L.M. Plank, R. Hewson); Vijayanagar Institute of Medical Sciences Bellay, Karnataka, India (A. Begum, M. Veera Shankar, R. Ravikumar); Royal Liverpool University Hospital, Liverpool (N.J. Beeching).

**Keywords:** chikungunya virus, central nervous system viral diseases, polymerase chain reaction, incidence, Japanese encephalitis vaccines, encephalitis, Japanese, dispatch

## Abstract

Chikungunya virus (CHIKV) is a mosquito-borne alphavirus best known for causing fever, rash, arthralgia, and occasional neurologic disease. By using real-time reverse transcription–PCR, we detected CHIKV in plasma samples of 8 (14%) of 58 children with suspected central nervous system infection in Bellary, India. CHIKV was also detected in the cerebrospinal fluid of 3 children.

Chikungunya virus (CHIKV) is a mosquito-borne alphavirus that causes illness characterized by fever, rash, and severe arthralgia. It was first described in Africa in 1952, and outbreaks occurred in India in the 1960s and early 1970s (*1*). Neurologic complications were reported occasionally (*2*,*3*). In 2005, an epidemic of CHIKV disease occurred among the populations of Réunion and other Indian Ocean islands (*1**,**4*)*,* and spread to India by early 2006, where an estimated 1.3 million persons were infected (*5*,*6*). During a prospective study of all children with suspected central nervous system (CNS) infections admitted to a hospital in rural southern India, we noticed an unseasonal increase in admissions. This increase occurred at the same time as the CHIKV outbreak in southern India, so we investigated our cohort for CHIKV infection.

## The Study

From January through October 2006, we studied children (<16 years of age) admitted to the pediatric department of the Vijayanagar Institute of Medical Sciences, Bellary, India, with suspected acute CNS infection. Acute CNS infections were suspected in those children with a febrile illness (<2 weeks’ duration) and 1 of the following signs or symptoms: meningism, photophobia, severe headache, altered mental status, seizures, or focal neurologic signs. Children with previous neurologic conditions or *Plasmodium falciparum* malaria were excluded. The study was approved by the ethical committees of the hospital, the Indian Council for Medical Research, and the University of Liverpool, United Kingdom. Informed consent was obtained from the accompanying parent or guardian.

A detailed history was taken, and a neurologic examination was performed by a member of the study team. Routine blood samples were collected, and a lumbar puncture was performed. To detect CHIKV RNA in the plasma or cerebrospinal fluid (CSF), real-time reverse transcription–PCR for a 127-base region of the envelope E1 gene was performed (*7*). The E1 gene was subsequently amplified by RT-PCR, and sequenced (*7*). A 529-base region of the sequence was aligned with other CHIKV E1 gene sequences by using Lasergene software (DNASTAR, Inc., Madison, WI, USA), and phylogenetic analysis was performed on the align sequences by using Mega 4 software (*8*). To detect antibodies against Japanese encephalitis (JE) virus and dengue virus, which circulate in this area, serum specimens and CSF were tested by immunoglobulin M (IgM) capture ELISA (*9*). PCR for JE virus was also performed on CSF samples (*10*).

From January 1 through October 31, 2006, 66 children were recruited for the study; 37 (56%) were male, median age was 7 years (range 8 months–16 years); 58 had at least 1 plasma sample, and 57 had a CSF sample available for testing. CHIKV was detected in 8 (14%) of the 58 plasma samples and in 3 (5%) of the CSF samples; CHIKV was not detected in 2 CSF samples, and no CSF samples were available for 3 children. The median (range 2–23) of days for a positive plasma sample was 3.5 days; the 3 positive CSF samples were all obtained within 4 days of illness onset (Appendix Table). Samples from all 8 patients were negative for malaria parasites and JE and dengue IgM antibodies. We also tested samples obtained before the outbreak (October 2005 through December 2005) and after the outbreak (November 2006 through December 2007); all were CHIKV negative.

Of the CHIKV-positive children, 7 children had altered mental status, which was associated with seizures in 6 patients; 3 children with both altered mental status and seizures also had meningism. Two children had a rash when they were hospitalized, and a rash developed in a third child on day 5 of hospitalization. Seven children had seizures and 4 had status epilepticus (seizure >30 min). Three children were aphasic and had extensor plantar reflexes. One 9-year-old girl (patient 5) had experienced 2 days of fever, vomiting, and a generalized tonic-clonic seizure at home that lasted 3 minutes; this occurred 3 days after she received a live attenuated SA14-14-2 JE vaccine. When hospitalized, she had a score of 15 on the Glasgow Coma Scale (GCS) and was monitored without a lumbar puncture. CHIKV was detected in her plasma. Only 2 of the 5 patients whose CSF was analyzed had pleocytosis (>5 cells × 10^9^/mL). Two children had reduced GCS scores, and 1 child remained aphasic when discharged. The other 6 patients were discharged with a full GCS score. At her 4-month follow-up visit, patient 8, who had CHIKV detected in her CSF and plasma, was performing poorly at school and had back and joint aches.

**Figure 1:**
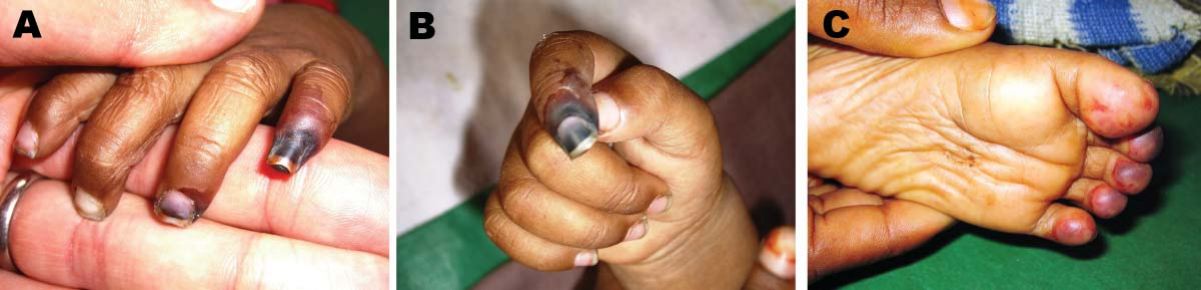
Digital gangrene in an 8-month-old girl during week 3 of hospitalization. She was admitted to the hospital with fever, multiple seizures, and a widespread rash; chikungunya virus was detected in her plasma. A) Little finger of the left hand; B) index finger of the right hand; and C) 4 toes on the right foot.

An 8-month-old girl (patient 7) was admitted who had experienced a fever for 7 days, multiple seizures, a widespread rash, and loss of appetite. She also had reduced hearing, a GCS score of 13, a vacant stare, frequent blinking, hepatomegaly (4 cm), and splenomegaly (6 cm). While she was an inpatient, gangrene developed in her fingers and toes (Figure 1). Her initial plasma and CSF samples were all used for clinical management, but a subsequent plasma sample was positive for CHIKV on day 23 of illness.

**Figure 2:**
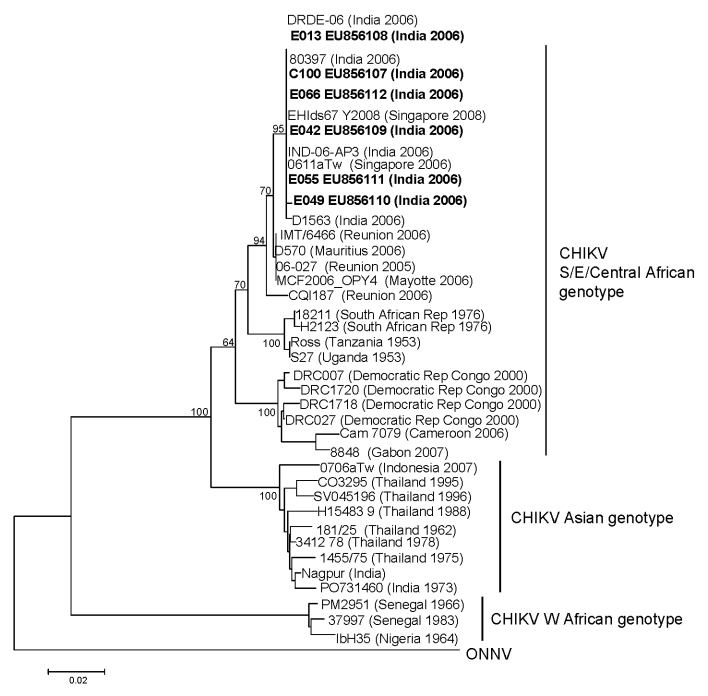
Phylogenetic analysis of chikungunya virus (CHIKV) sequences on the basis of partial E1 gene sequence (position 10620–11148 of the prototype CHIKV S27 genomic sequence). Sequences obtained in this study are in **boldface**. The analysis was performed using MEGA version 4 software (*8*), by using the neighbor-joining (p-distance) method. The length of the tree branches indicates the percentage of divergence; the percentage of successful bootstrap replicates is specified at the nodes (1,000 replicates). ONNV (o’nyong-nyong virus) prototype sequence was included to root the tree. Scale bar indicates number of nucleotide substitutions per site.

E1 gene PCR products sufficient for sequencing were amplified from plasma samples of 5 patients and the CSF of 1 patient. Sequences were deposited in GenBank under accession nos. EU856107–EU856112. Sequences were identical except for one that had a single nucleotide change from A to G at position 10625, resulting in an amino acid change from lysine to arginine at residue 211 of the E1 protein. The sequences from our cohort all had an alanine residue at position 226 of the E1 protein. This finding is typical of 90% of viral sequences from the Réuinion Islands from June 2005 through October 2005 (*11*). Isolates from the Réunion Island outbreak all had a valine at this position (*11*). Scientists have postulated that the change E1–A226V may be important for adaption to the mosquito vector, *Aedes albopictus,* and for neurovirulence (*11*–*13*). Our isolates lack this substitution. Phylogenetic analysis showed that the viruses were more closely related to those from the recent Indian Ocean CHIKV outbreaks and East African strains than to the Asian strains endemic in the region (Figure 2).

## Conclusions

Our study has confirmed that during a CHIKV outbreak, the virus may be an important cause of neurologic disorders in children. Recent studies have described a wide range of neurologic manifestations, including meningoencephalitis, seizures, and Guillain-Barré syndrome (*14*–*16*). Our study shows that CHIKV is a likely cause of CNS infection. During the outbreak period from January 2006 through October 2006, we found that CHIKV was responsible for 14% of suspected CNS infections. In 1 of our patients, an 8-month-old girl for whom no acute-phase sample was available, the virus was detected at day 23 of illness, an unusually persistent level of viremia. The severity of her illness, with marked rash, hepatosplenomgaly, and digital gangrene could have been due to her inability to clear the virus. Alternatively, she may have become infected with CHIKV during her hospital stay. If one excludes this patient from the analysis, CHIKV was detected in the plasma and CSF samples of 10% of patients with suspected CNS infection. Some children had other features suggestive of CHIKV infection, but in 4 case-patients, only neurologic symptoms were present. Notably, 1 child had received JE vaccine 3 days before admission as part of a mass JE vaccination campaign in India (*17*). Her illness was attributed to an adverse event after vaccination (*18*). We demonstrated that her illness was equally coincident with CHIKV infection, illustrating the importance of thorough investigation of cases of adverse events after vaccination.

In our study, we chose to rely on PCR detection of the virus to diagnose CHIKV infection rather than testing for IgM antibodies, which may persist for several months after infection and could reflect coincidental infection (*19*) rather than an acute infection. In summary, during CHIKV outbreaks, clinicians should be aware that CHIKV may be an likely cause of CNS infections among children.

## Supplementary Material

Appendix Table Clinical and laboratory features for 8 children with CNS infection due to chikungunya virus*
