# Supplementary material for: Chikungunya Virus and Central Nervous System Infections in Children, India
Source: Emerg Infect Dis. 2009 Feb;15(2):329–31. doi: 10.3201/eid1502.080902 (PMC2662654; doi:10.3201/eid1502.080902)
Supplement: Appendix Table — Clinical and laboratory features for 8 children with CNS infection due to chikungunya virus* [file 08-0902_appT-s1.pdf]

Appendix Table. Clinical and laboratory features for 8 children with CNS infection due to chikungunya virus*

				Clinical features on admission							
Patient no., age/sex	Day of illness	Rash	Vomiting	Headache	Meningism	No. seizures (status epilepticus, duration)	Altered mental status	GCS	Additional features	Laboratory investigations (day of illness)	Progress and outcome (day of illness)
1. 4 y/M	3	Yes	No	No	Yes	1 (yes, 2 h)	Yes	10 (E4, V2, M4)	Macular rash on trunk and lower limbs; aphasia, does not recognize anyone, wandering around; extensor plantars	CSF, WCC 8, (100% lymphocytes), protein 19.2; CSF glucose 109, blood glucose 86 (ratio 62%) CSF PCR† neg (D3); plasma PCR CHIKV** pos (D3)	Slow improvement, GCS 12 (E4, V3, M5) at discharge (D14)
2. 3 y/F	4	No	No	No	No	1 (No)	Yes	15 (E4, V5, M6)	Reduced feeding; equivocal plantars	CSF WCC 5 (100% lymphocytes), protein 16.2, CSF glucose, 86.7 CSF PCR pos CHIKV (D4); plasma PCR pos CHIKV (D4)	Good recovery, GCS 15 (D9)
3. 11 y/M	3	No	No	Yes	Yes	3 (Yes, 1 h)	Yes	9 (E2, V4, M3)	Conjunctivitis, drowsy, irritable, confused; extensor plantars	CSF sample for microbiology lost in transit CSF PCR CHIKV pos (D4); plasma PCR CHIKV pos (D4)	Day 5 of admission developed rash on trunk and face, GCS 15 (D11)
4. 5 y/M	3	No	No	Yes	Yes	1 (Yes, 1 h)	Yes	11 (E2, V4, M5)	Ear discharge, irritable; extensor plantars	CSF WCC 4 (100% lymphocytes), protein 24, CSF glucose 59; CSF PCR neg (D5); plasma PCR CHIKV pos (D5)	GCS 13 (E4, V4, M5) at discharge (D6)
5. 9 y/F	2	No	Yes	No	No	1 (No)	No	15 (E4, V5, M6)	Received SA14-14-2 live attenuated JE§ vaccine 3 days before admission	No CSF sample; plasma PCR CHIKV pos (D2)	GCS 15 (D3)
6. 8 y/M	5	No	Yes	Yes	No	0 (No)	Yes	12 (E3, V4, M5)	Irritable and confused; fixed stare, making abnormal sounds	CSF WCC 3 (100% lymphocytes), protein 42; CSF glucose 88, CSF PCR insuff; Plasma PCR CHIKV pos (D7)	Slow recovery to GCS 15 (D21)
7. 8 mo/M	7	Yes	No	No	No	10 (No)	Yes	13 (E4, V4, M5)	Petechiae over whole body, trunk and limbs; reduced feeding, reduced hearing bilaterally, vacant stare,	CSF WCC 125 (100% lymphocytes), protein 142, CSF glucose 30; CSF PCR insuff; plasma PCR CHIKV pos (D23)	Developed digital gangrene as inpatient; slow recovery to GCS 15 (D25)

									frequent blinking; hepatosplenomegaly		
8. 9 y/M	2	No	Yes	No	No	1 (Yes, 1 h)	Yes	10 (E2, V3, M5)	Aphasia, increased tone on left side	CSF WCC 5 (100% lymphocytes), protein 14.1, CSF glucose 95.6, CSF PCR CHIKV pos (D2); plasma PCR CHIKV pos (D2)	GCS 15 at discharge (D8); at 4 month follow-up, not doing as well at school, backache and joint ache

*CNS, central nervous system; E, eyes, V, verbal, M, motor; D, day of illness; JE, Japanese encephalitis; GCS, Glasgow Coma Scale; CSF, cerebrospinal fluid; CHIKV, chikungunya virus; WCC, white celll count; pos, positive; neg, negative; insuff., insufficient.
†Units for investigations: CSF WCC/mm3; CSF protein in mg/dL, CSF and blood glucose in mg/dL.
